# Notes and News

**Published:** 1891-09-05

**Authors:** 


### Notes and News.

Collected and Communicated.

Through the death of Dr. Sutton, late physician to the London Hospital, Dr. Turner, formerly assistant physician, has succeeded to that post. Dr. F. J. Smith, on Thursday last, was appointed assistant physician at a special Court held under Sir E. H. Currie's chairmanship.

---

At the monthly meeting of the Montrose Asylum and Infirmary Board it was stated that the Brechin District Committee had applied to the County Council to have a certain number of wards in Montrose Infirmary set aside for theuse of patients from the country, and an agreement making arrangements for the two small-pox wards in the Infirmary being placed at the disposal of the County Council, each ward having accommodation for ten or twelve patients, had been placed before the District Committee. For this privilege the County Council was to be asked to pay an annual sum of £50, and 5 per cent. on the cost of erecting a separate kitchen and washhouse which would be necessary. The County Council was also to pay the cost of nursing, medicine, &c., at such rates as might be fixed on by the Infirmary managers, who reserved power to accommodate only as many patients as they chose.

---

The *Globe* gives an interesting account of the Sailor's Home, Well's Street, a comfortable refuge for "Jack" at home. It stands surrounded by dock walls on one side and slums of the worst description on the other, making a kind of oasis amid a most dreary desert of vice and crime. Within its walls he feels himself comparatively safe, but outside the crimps and loafers stand like hungry wolves waiting to seize their prey. A few years ago, when a ship arrived in dock she was immediately boarded by a swarm of runners, and boarding house touts, all armed with bottles of spirits, which they distributed with the greatest liberality in the forecastles, making up to the men with soft speeches and strong rum, until they had persuaded them to go ashore in the most doubtful company. And thus Jack used to be led away like a sheep to the slaughter. Now, fortunately, things have changed, and no man need complain that temptation alone awaits him. When a ship arrives a cart from the Sailors' Home is waiting on the jetty, and an officer from the institution goes on board. All the men have then an opportunity of deciding whether they will go with him or not, and as a rule the whole crew avail themselves of his offer. The Home, which is quite self-supporting, is managed exceedingly well, and anyone who knows the dangers and misery that used to attend Jack when he only escaped the perils of the deep to fall into the pit of destruction at home, must be thankful for its presence in that dingy and desperate neighbourhood. There is sleeping accommodation for about six hundred men in the home, and seamen are boarded and lodged for fourteen shillings a-week. The board is very liberal and good, and consists of four meals a day.

At a special meeting of the Fellows of the Royal College of Surgeons, in their hall, Lincoln's Inn Fields, yesterday, to hear the report of the committee with regard to the proposed Albert University, the following resolution was passed: "That it would be inconsistent with the position and destructive of the functions of the Royal College of Surgeons of England to constitute itself any part of the Albert University for London; but in the event of the acceptance of a position in the Albert University by this college this meeting regards the representation accorded to the council of the new university as adequate.

---

The National Leprosy Fund, of which the Prince of Wales is President, publishes the journal of the Leprosy Investigation Committee, and communications on the subject from Norway, South Russia, Crete, the Cape of Good Hope, and New Brunswick. The *Daily Telegraph* remarks that it is strikingly illustrative of the difficulties which surround this subject that exactly opposite and equally confident answers are given to the question whether the disease is spread by contagion or is hereditary. Professor Münch is deeply convinced that the only cause of the development and spread of leprosy is contagion. On the other hand, Mr. Dixon, medical Superintendent of Robben Island, cites facts which "seem to indicate that lengthy and intimate personal contact is probably not the cause of the transmission of leprosy"; and Mr. Biliotti, writing in regard to Crete, considers it proved that the disease is not contagious, or only very slightly so.

---

In opening a school of ambulance at Woolwich, the Duchess of Teck has laid the foundation stone of a structure that has, at last, attained a solid basis. When "The St. John's Ambulance Classes" commenced their now widespread movement the subject was regarded with quite as much derision as approval. The whole scheme was regarded *dillitante*, and as dangerous as futile. "A little knowledge is a dangerous thing" was the byeword of those who looked askance at the movement. A vision of absurd scenes in which young ladies were tying up veins for arteries and arteries for veins, or distorting broken limbs, haunted the public mind. People needed to be reminded that any intelligent mind awakening tends to make the sensible more so, and that foolish persons would not be likely to be more foolish by knowing a little more. In the army, knowledge such as the ambulance societies spread is of untold assistance, and now that the measure is a recognised force the sphere of its benefits will increase rapidly.

---

The special meeting that was convened to discuss the question of sanitary progress in India, seems to have come to the conclusion that the progress has not been very great. Certainly a more appalling account than that which was given by Surgeon-Major Kirtikar and Mr. Ghole of the average sanitary condition of Indian towns and villages could not be well imagined. The water, upon which they are generally dependent for drinking and cooking purposes, is often filthy to the extent of being actually poisonous. The air of the small houses is so horribly vitiated that many of the natives fall victims to pulmonary consumption without having been touched by any hereditary taint. Ventilation and cleanliness in the streets and narrow alleys are simply non-existent, whilst cholera and fever are invited and encouraged by every condition of ordinary Indian life. The supervising sanitary officer of a large village is very often the lowest revenue officer of the district; probably a man without any special education, or, indeed, with very little education at all. As long as the initiative rests with such men reform is likely to be long in coming.

